# Challenging management of a baby with congenital multiple intestinal atresia, trisomy 18 and extremely low birth weight: a case report

**DOI:** 10.1186/s40792-023-01761-1

**Published:** 2023-10-13

**Authors:** Mitsumasa Okamoto, Sachiyo Fukushima, Satoshi Okada, Yudai Tsuruno, Hiroaki Fukuzawa, Tomoaki Ioroi, Masaaki Kugo

**Affiliations:** 1 Department of Pediatric Surgery, Himeji Red Cross Hospital, 1-12-1, Shimoteno, Himeji, Hyogo 670-8540 Japan; 2 Department of Neonatology, Himeji Red Cross Hospital, 1-12-1, Shimoteno, Himeji, Hyogo 670-8540 Japan

**Keywords:** Congenital multiple intestinal atresia, Extremely low birth weight, Life prognosis, Trisomy 18

## Abstract

**Background:**

Extremely low birth weight (< 1000 g) still influences postsurgical prognosis in the neonatal and infantile periods. Additionally, the life expectancy of neonates with trisomy 18 is extremely poor owing to various comorbidities. Therefore, it takes courage to perform laparotomy for the purpose of treatment of congenital multiple intestinal atresia in a baby with an unpredictable life prognosis.

**Case presentation:**

Fetal ultrasonography revealed cardiac malformation, intestinal dilation, and physical characteristics suggestive of a chromosomal abnormality in this case. The patient was diagnosed with trisomy 18 after birth, with an extremely low birth weight. An atrial septal defect, ventricular septal defect, dilated jejunum, and a very thin collapsed small intestine were found on ultrasonography. With a diagnosis of congenital small intestinal atresia, a challenging laparotomy was done at 3 days of age, with jejunal atresia and multiple distal small intestinal atresia were observed. The jejunal end and distal small intestinal stump were separated into stomas at the wound edge. Hypertrophic pyloric stenosis developed at the age of 3 months and resolved with medication. The patient gained weight (2 kg) by daily stool injection into anal side of the intestine and decompression against poor peritonitis of dilated jejunum using enteral feeding tube for the long period. Finally, we could perform intestinal reconstruction safely and successfully at the age of 9 months.

Tracheotomy was performed due to difficulty in extubation associated with chronic lung disease. The patient was discharged at the age of 1 year and 3 months, and no major problems were noted at the age of 2 years.

**Conclusions:**

We treat congenital intestinal atresia in extremely low birth weight infants with severe chromosomal abnormalities and severe cardiac malformations as follows: Stoma creation is performed quickly to avoid deterioration of the patient's hemodynamics. After that, while continuing enteric management, palliative cardiovascular surgery is performed as necessary, and the patient's body weight and intestinal tract status are determined to allow safe intestinal reconstruction.

## Background

Extremely low birth weight (ELBW) influences the life prognosis after surgery in the neonatal and infantile periods [1, 2]. Additionally, the life expectancy of neonates with trisomy 18 is extremely poor due to various comorbidities [3]. Surgeries of congenital intestinal atresia in the baby with these two critical problems are “challenging” procedures and long-term intensive care is also indispensable for survival. Therefore, management challenges of this patient are worth reporting in order to aid in the treatment of similar patients in the future.

## Case presentation

**Fig. 1:**
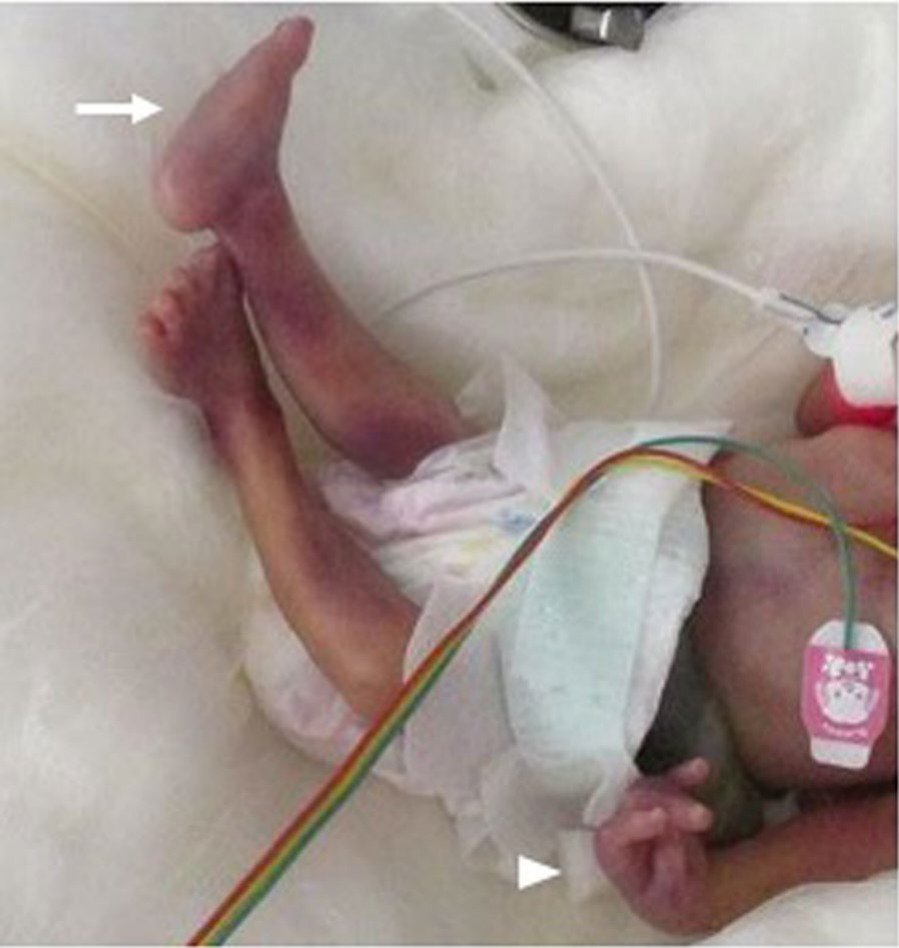
Appearance of the patient soon after birth. Rocker bottom foot (arrow) and overlapping finger (arrowhead) are observed

A male baby with a gestational age of 29 weeks and 6 days was delivered by emergency cesarean section with a birth weight of 699 g because of HELLP syndrome. Fetal ultrasonography revealed cardiac malformation, intestinal dilation, and physical characteristics suggestive of a chromosomal abnormality. After birth, rocker’s bottom foot and overlapping fingers were observed (Fig. 1).

**Fig. 2:**
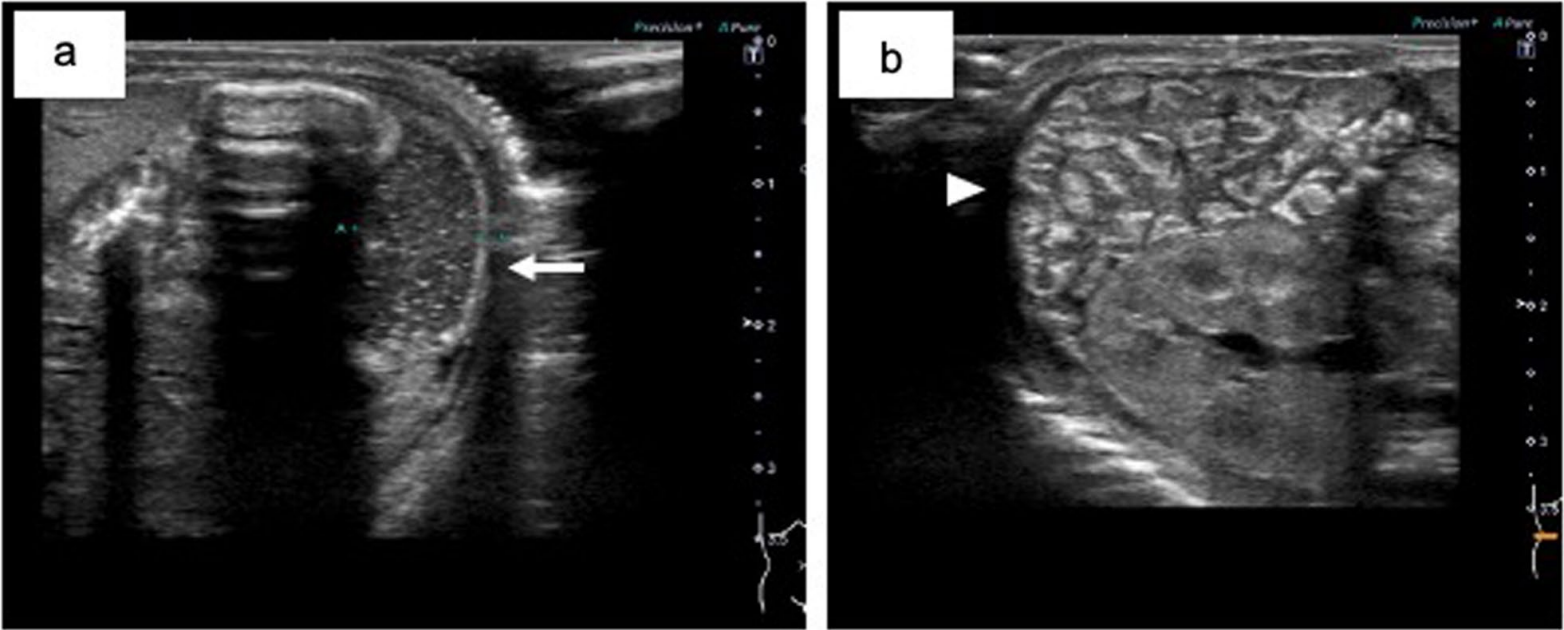
Abdominal ultrasonography findings. A dilated jejunum (arrow) (**a**) and collapsed small intestine (arrowhead) (**b**) are seen

The patient was diagnosed with trisomy 18 based on G-band karyotyping. Atrial septal defects (ASD) and ventricular septal defects (VSD) were identified using echocardiography. The dilated jejunum and collapsed small intestine were detected using abdominal ultrasonography (Fig. 2).

**Fig. 3:**
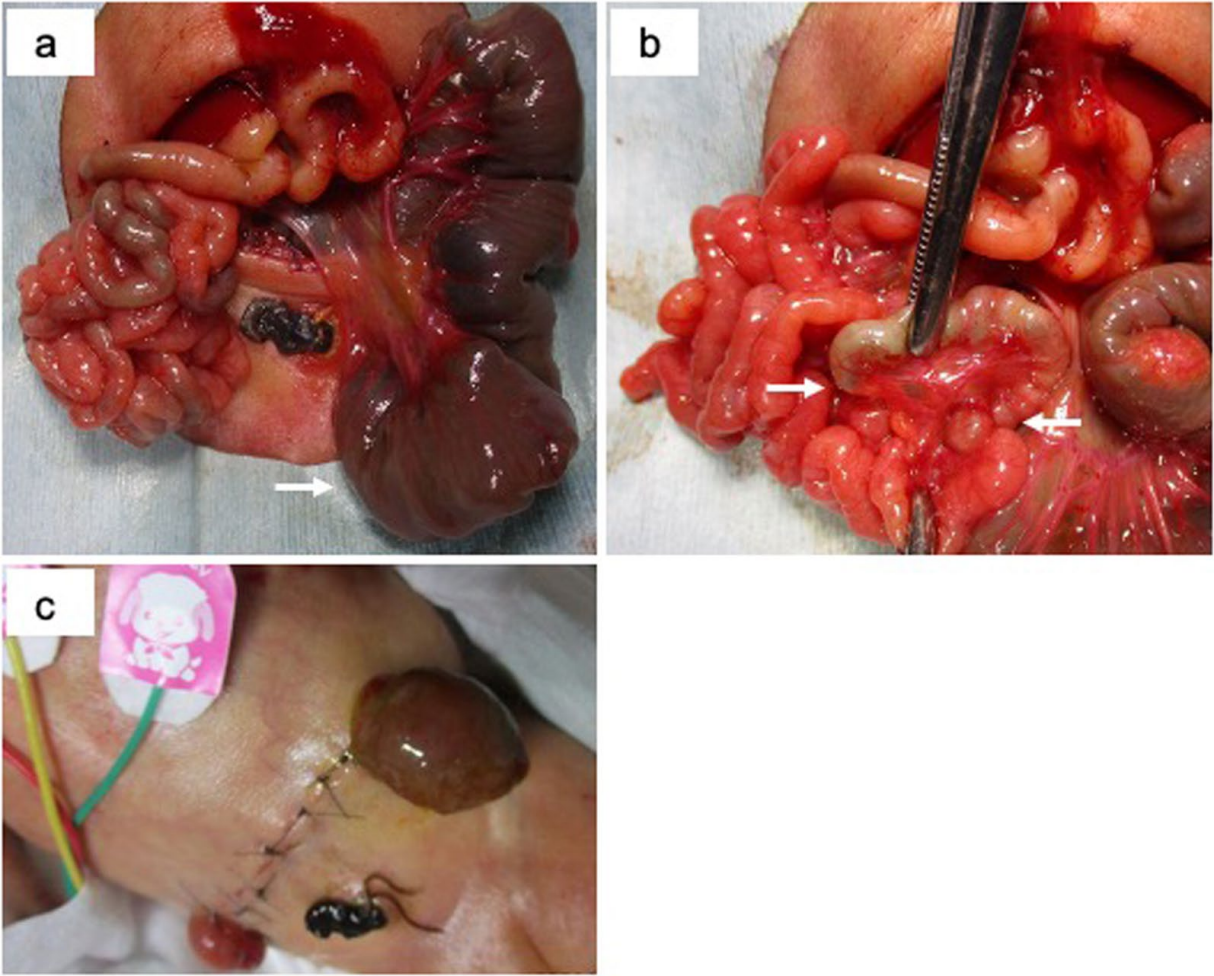
Intra and post operative views. Arrows indicate atretic points of the jejunum and the distal small intestine (**a**, **b**)

With a preoperative diagnosis of congenital jejunal atresia, we decided to perform a laparotomy at 3 days of age after discussing the patient’s life prognosis with the family and NICU staff. Jejunal atresia (Fig. 3a) and multiple distal small intestinal atresia (Fig. 3b) were also observed. Because of the large difference in diameter between the jejunum and small intestine (10 mm vs. 2 mm), it was determined that one-stage anastomosis would be difficult. The jejunal end and distal small intestinal stump were separated into stomas at the wound edge (Fig. 3c).

**Fig. 4:**
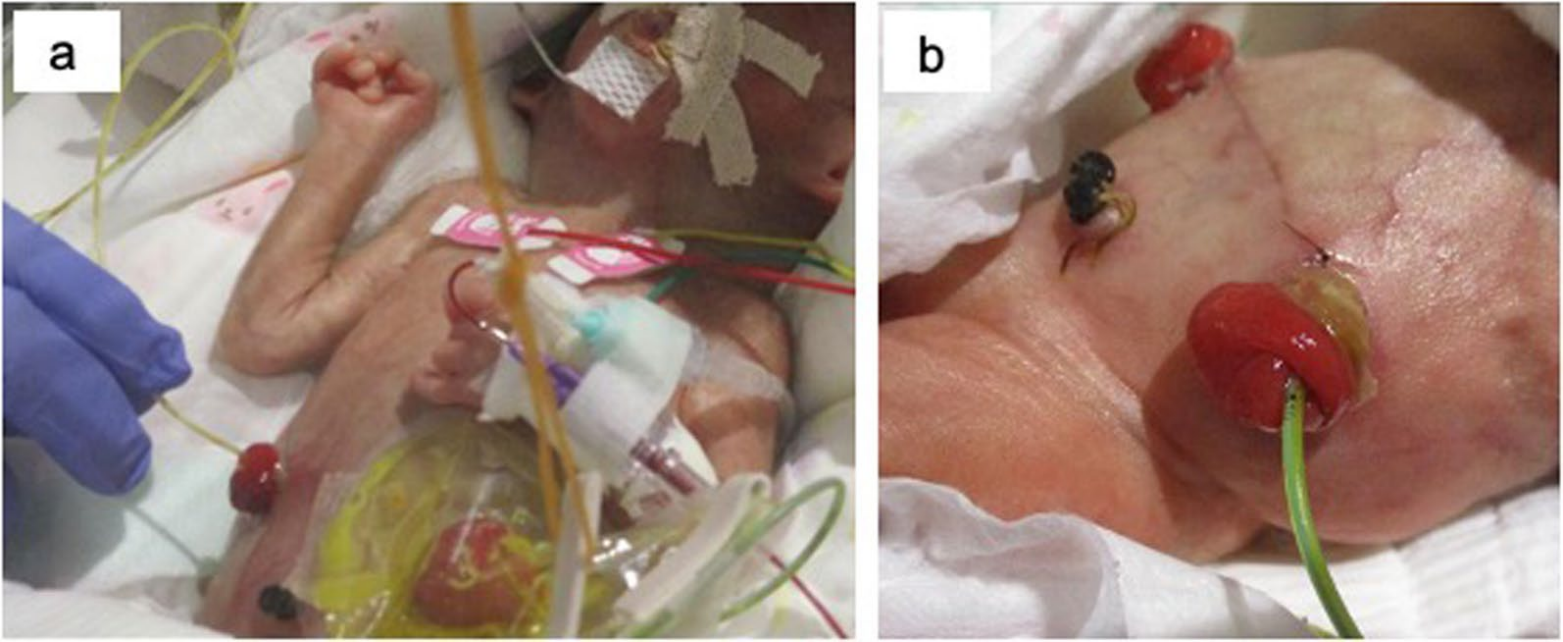
Intestinal management after stoma construction. Stool injection into the distal small intestine (**a**) and stool discharge using an enteral feeding tube (**b**) were continued until intestinal reconstruction was performed

Milk feeding via nasogastric tube and stool injection into the distal small intestine continued until intestinal reconstruction was performed (Fig. 4a). Since poor peristalsis of the jejunum was observed one month after enterostomy, a 5-Fr enteral feeding tube was inserted all the time through the jejunal stoma to improve stool discharge (Fig. 4b) [4].

**Fig. 5:**
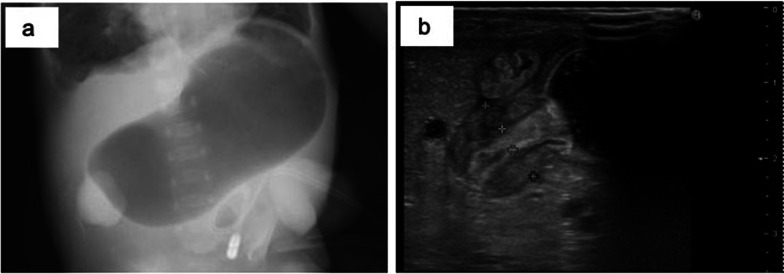
Abdominal radiography and ultrasonography findings at onset of hypertrophic pyloric stenosis. Distended stomach (**a**) and pyloric muscle thickened to 4-5 mm (**b**) are seen

At 3 months of age, hypertrophic pyloric stenosis (HPS) was developed (Fig. 5) and resolved by atropine sulfate intravenous administration (0.03–0.08 mg/kg/day) for 2 months, without significant side effects such as tachycardia [5].

Consequently, intestinal reconstruction was delayed until body weight gain was achieved.

**Fig. 6:**
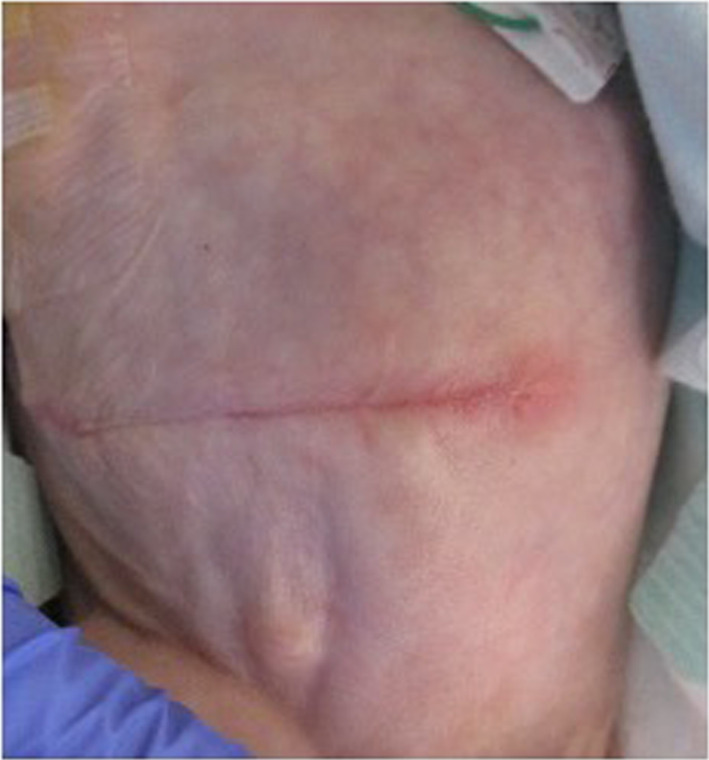
Wound appearance one month after surgery. Surgery scars are less noticeable

When his body weight reached 2 kg, intestinal reconstruction was performed safely and successfully at the age of 9 months (Fig. 6).

He underwent tracheotomy due to difficulty in extubation associated with chronic lung disease, and despite careful surgery, lymphorrhoea developed immediately after surgery and required sedation for several days. Eventually he was discharged from the hospital at the age of 1 year and 3 months. He has been living using a home respirator without any major problems.

## Discussion

Recent advances in perinatal and neonatal care have increased survival rates of infants admitted to the neonatal intensive care unit (NICU), but extremely low birth weight (ELBW) or extremely lower operative weight is still associated with postoperative mortality [1, 2]. Regarding the prognosis of congenital intestinal atresia, mortality rates for all neonates are low (~ 5%) [6, 7], but rates of ELBW infants are 7/19: relatively high in Japan [8]. Furthermore, the life prognosis of trisomy 18 neonates is very poor due to various comorbidities, including congenital heart disease, and reportedly, the 1-year survival rate is 5–10% [3]. Esophageal atresia and intestinal malrotation are frequently observed in gastrointestinal malformations of trisomy 18 [9], but complications with intestinal atresia are extremely rare.

Association of HPS with Trisomy 18 has been previously reported [9]. We suspected it would be difficult to conduct simultaneous pyloromyotomy and reconstruction of small intestine “safely” because physical growth was not sufficient, therefore, we chose medical therapy. In addition, because the effect of intragastrointestinal administration of atropine sulfate was unclear in the small intestine, intravenous administration was performed throughout the study period [5].

We wondered whether surgery should be performed for an extremely low birth weight neonate with trisomy 18, because he may suffer from severe mental retardation and require extensive nursing care even if he survives long term.

After discussing with the patient’s family and NICU staff, we decided to perform a laparotomy to preserve the patient's life because the VSD and ASD were not symptomatic.

A high survival rate was described for extremely low birth weight infants with congenital small intestinal atresia by primary anastomosis [8]. However, we were concerned that the small caliber of the anal side of the intestine due to physical immaturity and the widening of the caliber ratio of the anastomotic intestine due to dilation of the proximal side of the intestine might be the risk of dysfunction of anastomotic site. Therefore, we performed stoma creation and continue enteric management to solve the problems of body weight gain and the caliber difference. And at the body weight of the patient was 2 kg, we decided to perform intestinal reconstruction and succeeded safely.

Although there is some discussion to limit life-sustaining treatment for neonates with trisomy 18 [10], we opted to practice intensive and safe treatments, including intestinal managements after the construction of the stoma for the long time. In this case, VSD and ASD were not symptomatic. If palliative treatment for cardiac malformations is required (for example PA banding), we consider it is better to perform intestinal reconstruction after cardiac surgery to stabilize hemodynamics so that intestinal reconstruction can be performed safely. This decision was based on the judgement that individual management policies should consider the patient’s condition and family opinions [3, 11].

## Conclusions

Here, we encountered a rare case of trisomy 18 in a patient with ELBW, multiple congenital small intestinal atresia, and congenital heart malformations. We treat congenital intestinal atresia in extremely low birth weight infants with severe chromosomal abnormalities and severe cardiac malformations as follows: Stoma creation is performed quickly to avoid deterioration of the patient's hemodynamics. After that, while continuing enteric management, palliative cardiovascular surgery is performed as necessary, and the patient's body weight and intestinal tract status are determined to allow safe intestinal reconstruction. The family's opinion should also be considered.

## Data Availability

Not applicable.

## Abbreviations

ASD: Atrial septal defect
ELBW: Extremely low birth weight
HPS: Hypertrophic pyloric stenosis
NICU: Neonatal intensive care unit
VSD: Ventricular septal defect
